# Supplementary material for: Towards unified AI-driven fracture mechanics: the extended deep energy method (XDEM)
Source: Nat Commun. 2026 Aug 15;17:8492. doi: 10.1038/s41467-026-76748-1 (PMC13484471; doi:10.1038/s41467-026-76748-1)
Supplement: Supplementary file 3 [file 41467_2026_76748_MOESM3_ESM.zip › pic/Results/Crack_propagation/Straight/3D_dis_force/pic/refined_points.pdf]

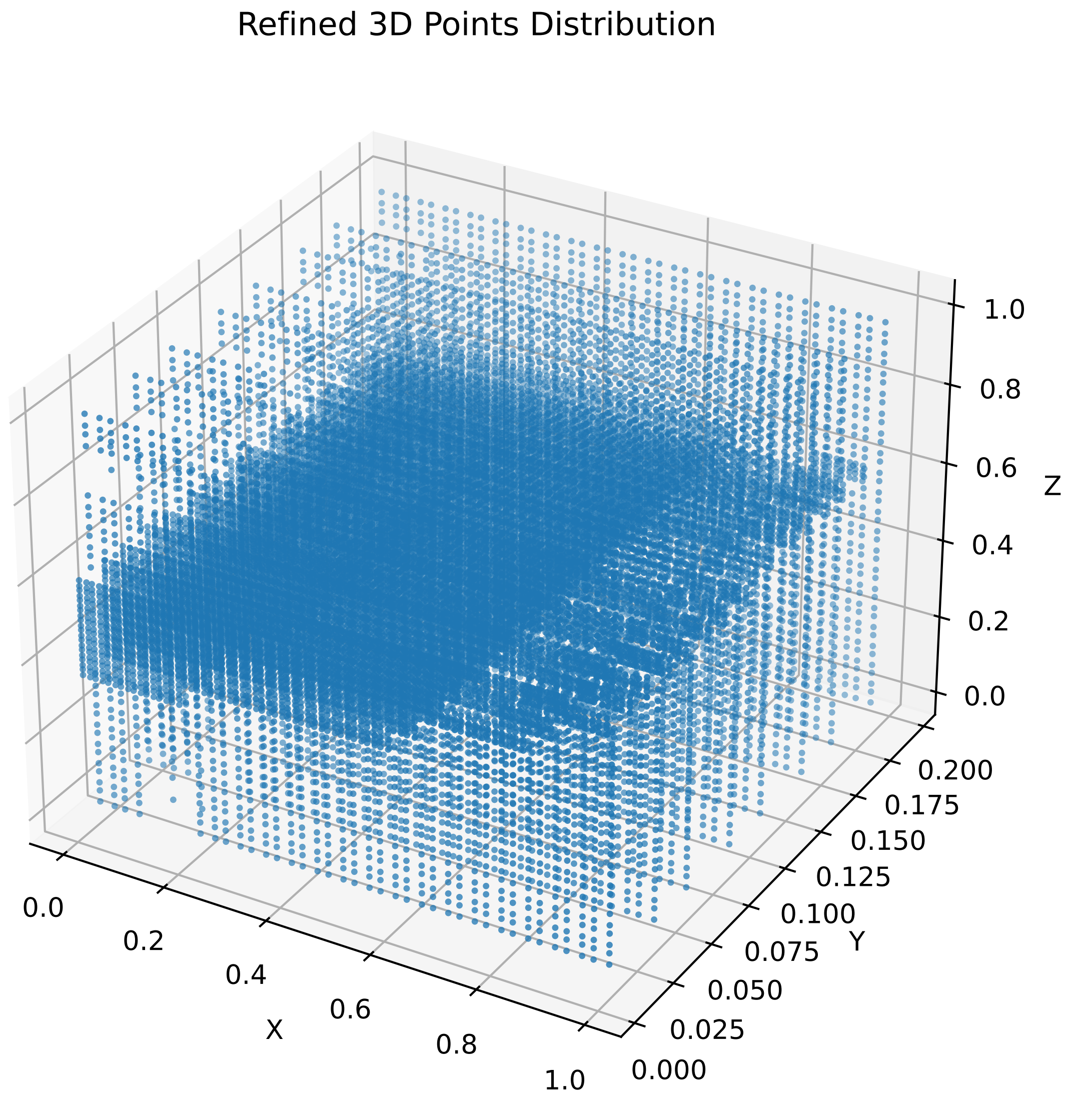

Refined 3D Points Distribution
X
Y
Z
0.0
0.2
0.4
0.6
0.8
1.0
0.000
0.025
0.050
0.075
0.100
0.125
0.150
0.175
0.200
0.0
0.2
0.4
0.6
0.8
1.0
